# A School-Based Study with Rome III Criteria on the Prevalence of Functional Gastrointestinal Disorders in Chinese College and University Students

**DOI:** 10.1371/journal.pone.0054183

**Published:** 2013-01-18

**Authors:** Yan-Yan Dong, Fei-Xue Chen, Yan-Bo Yu, Chao Du, Qing-Qing Qi, Han Liu, Yan-Qing Li

**Affiliations:** 1 Department of Gastroenterology, Qilu Hospital, Shandong University, Jinan, Shandong, People’s Republic of China; 2 The Key Laboratory of Cardiovascular Remodeling and Function Research, Chinese Ministry of Education and Chinese Ministry of Health, Qilu Hospital, Shandong University, Jinan, Shandong, People’s Republic of China; Federal University of Rio de Janeiro, Brazil

## Abstract

**Background:**

Functional gastrointestinal disorders, including functional dyspepsia, irritable bowel syndrome and functional constipation are very common worldwide.

**Objective:**

This research aims to estimate the prevalence and associated factors involved in functional gastrointestinal disorders in Chinese college and university students using the Rome III criteria.

**Methods:**

A total of 5000 students from Shandong University in China were asked in January-May 2012 to complete questionnaires, including the Rome III questionnaire, hospital anxiety and depression scale, and negative life events scale.

**Results:**

Based on the 4638 students who completed the questionnaire, the prevalence of functional dyspepsia, irritable bowel syndrome and functional constipation in college and university students of North China worked out to be 9.25%, 8.34% and 5.45% respectively. They were more frequent in female students. The factors of anxiety (OR 1.07; 95% CI 0.99 to 1.16, P = 0.002<0.05) and depression (OR 0.55; 95% CI 0.15 to 1.05, P = 0.045<0.05) indicated a high risk of causing irritable bowel syndrome.

**Conclusion:**

Functional dyspepsia, irritable bowel syndrome and functional constipation were common in college and university students of North China. Psychological disorders such as anxiety and depression provide significant risk factors for irritable bowel syndrome patients.

## Introduction

Functional gastrointestinal disorders (FGIDs) are very common worldwide [1]. They have a negative impact on health-related quality of life and result in high health care expenditures [2]. The Rome III criteria of FGIDs were updated from the Rome II criteria in 2006 [3]. There are few studies that have estimated the prevalence and associated factors of FGIDs in selected populations using Rome III criteria [4].

Functional dyspepsia (FD), otherwise known as non-ulcer dyspepsia, is clearly the most common cause of dyspeptic symptoms in the upper abdomen [5]. FD is divided into two subgroups according to the Rome III criteria: epigastric pain syndrome (EPS) and postprandial distress syndrome (PDS) [6]. The frequency of FD varied from 8%–23% of the population [7], [8]. A similar survey conducted in Korea using Rome III criteria indicated a frequency 8.1% in community population [9]. Most patients with FD complain of several symptoms related to meals. However, the pathophysiology of this disease remains poorly defined.

Irritable bowel syndrome (IBS) is a common, costly and functional gastrointestinal disorder characterized by abdominal pain or discomfort with altered bowel habits, but without any organic damages such as tumor or inflammation [3]. The prevalence of IBS is 15%–24% in the general population of Western countries, while the rate is 5%–10% in Asia [10]–[12]. It has been shown that some factors, especially psychological factors, such as anxiety and depression, are associated with the onset and course of IBS [13].

Functional constipation (FC) is a common type of FGIDs that manifests itself as persistently difficult, infrequent, or incomplete defecation, which does not meet the IBS criteria [3]. A survey showed the prevalence of FC in the general population of United States was 14.7% [14].

This study aimed to estimate the prevalence and associated factors involved in functional gastrointestinal disorders in Chinese college and university students using the Rome III criteria.

## Materials and Methods

### Ethics Statement

All participants gave their written informed consent prior to data collection. The study was approved by the Ethics Committee of Qilu Hospital of Shandong University, China.

### Study Setting

This investigation was carried out from January to May 2012 at Shandong University, located in Northern China.

### Sample Size

It has been generally reported that the prevalence of FD, IBS and FC is 8.1%, 10%–25%, and 14.7% of the population, respectively. According to the established formula, the sample size of 5000 enrolled in this research is sufficient to achieve a precision of ±2% with a 95% confidence interval (CI) [15].

### Participants

A total of 5000 college and university students, randomly recruited from 3 areas of study (liberal arts, science, and medicine), were asked to voluntarily complete questionnaires. Their age span from 18 to 23 years old. Trained staff answered any questions on the spot.

### Measures

#### Chinese version of Rome III questionnaire

The Rome III criteria have been widely used for the diagnosis of functional gastrointestinal disorders. This self-report standard questionnaire is developed by the Rome Foundation Board to identify FGIDs using the Rome III criteria [3]. This questionnaire has been repeatedly tested and very carefully validated [16]. We used the Chinese version of the previously validated Rome III diagnostic questionnaire. During the past years, the Chinese version has been extensively validated in a variety of adolescents and adult populations, including clinical practice and community research, with well-documented good measurement properties [17]–[19].

#### Diagnostic criteria of FGIDs

A Rome III diagnostic criterion of FD required one or more of the following symptoms: (1) bothersome postprandial fullness, (2) early satiation, (3) epigastric pain, and (4) epigastric burning. And no evidence of structural diseases (including on upper gastrointestinal endoscopy) that is likely to explain the symptom was necessary. Diagnostic criteria of subtype PDS must include one or both of the following: (1) bothersome postprandial fullness, happening after ordinary-sized meals, at least several times per week and (2) early satiation that prevents finishing a regular meal, at least several times per week. Diagnosis criteria of subtype EPS must include all of the following criteria: (1) pain or burning localized to the epigastrium of at least moderate severity, at least one time per week, (2) the pain to be intermittent, (3) not generalized or localized to other abdominal or chest regions, (4) not relieved by defecation or passage of flatus, and (5) neither criteria for gallbladder nor sphincter of Oddi disorders. All the above criteria fulfilled for the last 3 months with symptom onset at least 6 months prior to FD diagnosis.

To diagnose IBS, patients must have recurrent abdominal pain or discomfort for at least 3 months in the previous 6 months, with 2 or more of the following symptoms: (1) relief with defecation, (2) onset associated with a change in frequency of stool, and (3) onset associated with a change in form (appearance) of stool. The classification of IBS subtypes was based on the predominant stool pattern. IBS with constipation (IBS-C) was defined as having hard or lumpy stool at least 25% of the time and loose (mushy) or watery stools less than 25% of bowel movements. IBS with diarrhea (IBS-D) diagnosis was defined as having loose (mushy) or watery stools at least 25% of the time and hard stools less than 25% of bowel movements. And mixed IBS (IBS-M) was defined as having hard or lumpy stool at least 25% of bowel movements and loose (mushy) or watery stool at least 25% of bowel movements. Unsubtyped IBS means insufficient abnormality of stool consistency to meet criteria other three subtypes.

FC was diagnosed as the presence of defecation discomfort during at least 3 months in past 6 months with 2 or more of these following symptoms: (1) Straining during at least 25% of defecation; (2) Lumpy or hard stools in at least 25% of defecation; (3) Sensation of incomplete evacuation for at least 25% of defecation; (4) Sensation of anorectal obstruction or blockage for at least 25% of defecation; (5) Manual maneuvers to facilitate at least 25% of defecation (e.g. digital evacuation, support of pelvic floor); (6) Fewer than three defecation per week. And (1) loose stools are rarely present without the use of laxatives, (2) insufficient criteria for irritable bowel syndrome are necessary [3].

#### Hospital anxiety and depression scale (HADS)

The hospital anxiety and depression scale (HADS), a specifically reliable scale developed by Zigmond and Snaith [20], can be used in estimating the emotional disorders of anxiety and depression symptomatology in IBS sufferers. It has published for a long time and is a strong clinical measure that is well validated [21], [22]. The good reliability and stability of the HADS were demonstrated in Chinese translated versions [23]–[25]. The HADS is a short, self-reporting questionnaire consisting of 14 questions with two 7-item subscales for anxiety and depression assessment. For each subscale, the scores can be divided into 0–7 (normal cases), 8–10 (borderline cases), and over 11 (severe cases). Moreover, anxiety and depression scores are summed separately.

#### Red-flag items

Referring to the guidelines for FGIDs recommended by the American Gastroenterological Association, a series of “red flag” or alarm symptom questions used to distinguish structural intestinal disease from FGIDs [3]. They include drastic weight loss, a history of organic bowel disease, a history of digestive surgery, blood in stool, awakening due to abdominal pain during night, anemia, fever or arthralgia. Participants who were identified with one or more of these 7 items were excluded from the survey.

#### Negative life events in childhood

A life events scale was used in this study focusing on IBS. It has been used in two surveys involving large populations [26], [27]. Considering the specific situations of college and university students in China, we assessed 5 of the most stressful events in childhood (occurring under the age of 12 years) in the scale. These included the death of a close family member, divorced parents, extreme financial difficulties, experiencing serious illness or major surgery, major natural disaster or other serious setbacks. The presence of each event was totaled (range 0–5). It was divided it into 0 (normal cases), 1–2 (mild cases), 3–4 (moderate cases), and 5 (severe cases).

#### Baseline information

The remaining questions are about the socio- demographic characteristics, such as age, sex, schooling level, place of birth, health condition, and personal family information.

### Statistical Analysis

All eligible questionnaires were coded. Data analysis was performed using the Statistical Package for Social Science Software for Windows (SPSS 15.0 version). All calculated P-values were two-tailed and P<0.05 was considered statistically significant. Analysis of variance was used to compare the anxiety, depression and negative life events levels between groups. Possible risk factors were assessed by logistic regression analysis. Odds ratio (OR) with 95% confidence interval (CI) was calculated. Data are presented as mean ± SD.

## Results

### Response Rate and Characteristics of Subjects

Of the 5000 enrolled participants, 4860 completed the questionnaires. Valid responses were obtained from 4638 participants, with a response rate of 92.76%. Of the valid 4638 participants, 2215 (47.8%) were males and 2423 (52.2%) were females, with a mean age of 20.64±1.428 years and 20.88±1.572 years, respectively. The average college level of the students was 2.53±0.162. Of the valid 4638 participants, 3461 (74.6%) were natives of Shandong Province. The characteristics of participants are listed in Table 1.

**Table 1:** Characteristics of students with functional gastrointestinal disorders.

	Total(n = 4638)	Normal (%)(n = 3581)	FD (%)(n = 429)	IBS (%)(n = 387)	FC (%)(n = 253)
Gender					
Male	2215 (47.8)	1767 (49.3)	192 (44.8)	176 (45.5)	84 (33.2)
Female	2423 (52.2)	1814 (50.7)	237 (55.2)	211 (54.5)	169 (66.8)
Branch of learning					
Liberal arts	1637 (35.3)	1236 (34.5)	160 (37.3)	147 (38.0)	101 (39.9)
Science	1546 (33.3)	1199 (33.5)	132 (30.8)	125 (32.3)	93 (36.8)
Medicine	1455 (31.4)	1146 (32.0)	137 (31.9)	115 (29.7)	59 (23.3)
School Level					
Lower	2950 (63.6)	2279 (63.6)	278 (64.8)	238 (61.5)	162 (64.0)
Upper	1688 (36.4)	1302 (36.4)	151 (35.2)	149 (38.5)	91 (36.0)

Data are number (%).

FD: Functional Dyspepsia;

IBS: Irritable Bowel Syndrome;

FC: Functional Constipation.

### Prevalence of FGIDs

Of the valid 4638 participants, 429 fulfilled criteria for FD, while 387 fulfilled for IBS with a male/female ratio of 1∶1.20, and 253 had FC. The prevalence of FD, IBS and FC was 9.25%, 8.34% and 5.45% respectively, with no statistically significant difference among areas of study or schooling level. The detailed FD and IBS subtype characteristics are described in Table 2. Twelve students had both FD and IBS disorders. Participants who fulfilled the criteria of FC were classified according to their symptoms (Table 3).

**Table 2:** Characteristics of students with functional dyspepsia and irritable bowel syndrome subtypes.

	FD(n = 429)	PDS (%)(n = 265)	EPS (%)(n = 164)	IBS(n = 387)	IBS-C (%)(n = 150)	IBS-D (%)(n = 189)	IBS-M (%)(n = 48)
Gender							
Male	192	122 (63.5)	70 (36.5)	176	66 (37.5)	92 (52.3)	18 (10.2)
Female	237	143 (60.3)	94 (39.7)	211	84 (39.8)	97 (46.0)	30 (14.2)
Branch of learning							
Liberal arts	160	87 (54.4)	73 (45.6)	147	63 (42.9)	65 (44.2)	19 (12.9)
Science	132	80 (60.6)	52 (39.4)	125	50 (40.0)	60 (48.0)	15 (12.0)
Medicine	137	98 (71.5)	39 (28.5)	115	37 (32.2)	64 (55.6)	14 (12.2)
School Level							
Lower	278	173 (62.2)	105 (37.8)	38	96 (40.3)	104 (43.7)	38 (16.0)
Upper	151	92 (60.9)	59 (39.1)	149	54 (36.2)	85 (57.1)	10 (6.7)

Data are number (%).

FD: Functional Dyspepsia;

IBS: Irritable Bowel Syndrome.

**Table 3:** Description of symptoms for students with functional constipation.

Symptoms	Male (%)	Female (%)	Total
Straining	81 (96.4)	156 (92.3)	237 (93.7)
Lumpy or hard stools	74 (88.1)	150 (88.8)	224 (88.5)
Incompleteevacuation	75 (89.3)	165 (97.6)	240 (94.9)
Anorectal obstruction	72 (85.7)	150 (88.8)	222 (87.7)
Manual maneuvers	36 (42.9)	83 (49.1)	119 (47.0)
Fewer defecation	82 (97.6)	167 (98.8)	249 (98.4)

Data are number (%).

FC: Functional Constipation.

### HADS Scores and Negative Life Events in Childhood of IBS

The hospital anxiety and depression scores were significantly higher for students with IBS (Table 4), especially for those with normal scores (0–7) than for those with abnormal scores (>11). The mean anxiety score were 6.23±4.564 (IBS-C), 6.65±4.934 (IBS-D), and 6.47±4.360 (IBS-M). The mean depression score were 6.76±3.946 (IBS-C), 6.69±4.362 (IBS-D), and 6.96±4.658 (IBS-M). No difference was found in anxiety and depression scores between the 3 subtype groups. No significant difference was found due to negative life events in childhood.

**Table 4:** Univariate analysis of psychological factors of irritable bowel syndrome and control groups.

Factors		Total(n = 3968)	Normal (%)(n = 3581)	IBS (%)(n = 387)	χ^2^	*P* value
Anxiety						
	0–7	3108 (78.3)	2871 (92.4)	237 (7.6)	98.80	0.00
	8–10	552 (13.9)	435 (78.8)	117 (21.2)	3.67	0.06
	≥11	308 (7.8)	275 (89.3)	33 (10.7)	15.08	0.00
Depression						
	0–7	2827 (71.2)	2611 (92.4)	216 (7.6)	208.86	0.00
	8–10	216 (5.4)	134 (62.0)	82 (38.0)	3.66	0.06
	≥11	925 (23.3)	836 (90.4)	89 (9.6)	110.40	0.00
Negative life events in childhood						
	Normal	2025 (51.0)	242 (12.0)	1783 (88.0)	6.58	0.01
	Mild	1306 (32.9)	105 (8.0)	1201 (92.0)		
	Moderate	558 (14.1)	38 (6.8)	520 (93.2)		
	Severe	79 (2.0)	2 (2.5)	77 (97.5)		

Data are number (%). IBS: Irritable Bowel Syndrome.

### Risk Factors for IBS

After the univariate analysis, the multivariable logistic regression analysis was adjusted by sex type. The factors of anxiety (OR 1.07; 95% CI 0.99 to 1.16, P = 0.002<0.05) and depression (OR 0.55; 95% CI 0.15 to 1.05, P = 0.045<0.05) were independently associated with IBS (Table 5).

**Table 5:** Multivariate logistic regression to evaluate psychological factors of IBS.

Variables	Beta	Standard Error.	Wald	OR (95% CI)	P value
Anxiety	0.072	0.023	10.040	1.07 (0.99 to 1.16)	0.002
Depression	−0.605	0.301	4.034	0.55 (0.15 to 1.05)	0.045

## Discussion

FGIDs have become prevalent worldwide in recent years [10], [11]. Few data has been shown on the systematic epidemiology of FGIDs in student population. Considering as a young adult community, college and university students in China have similar diet, life activity, educational situation and so on. To our knowledge, the present study is the largest population school-based investigation on the epidemiology of FGIDs in college and university students. The results of the present study demonstrate that the prevalence of FD, IBS and FC was found to be 9.25%, 8.34% and 5.45% respectively, with more female students than male students suffering from FGIDs.

The prevalence of FD varied greatly in the general population [7]–[9]. We found that PDS was the more common subtype compared to EPS and this finding compared well with similar outside research [4], [28]. The reason is thought to be that the diagnostic criteria of EPS are too strict and make it difficult to distinguish between heartburn and epigastric burning in the questionnaire procedure [4]. Most of these studies have limited number of population. Our study showed more detailed epidemiological data.

Our research showed the epidemiological rate of IBS in college and university students was 8.34%. The prevalence of IBS in community population varied greatly among different investigations. About 10% to 20% of adults have IBS symptoms, and this disorder affects all races throughout the world [29]–[31]. It suggested that may be due to the different diagnostic criteria such as Rome II and Rome III used. For example, a research in Southeast China assessing the prevalence of IBS among undergraduates reported 4.7% and 10.4% using Rome II and Rome III criteria respectively [18]. Studies suggest that the Rome III criteria are based on a lower frequency and shorter duration of IBS symptoms than the Rome II criteria [32]. Rome II criteria have been used to examine a 12-week period duration in the past 12 months, which is less than a continuous 6-month period, thus expanding the scope of diagnosis and were stricter than the Rome III criteria [33].

In our study, the female/male ratio of IBS was 1.20∶1. Gender differences in IBS may be due to several factors including differences in biobehavioral responses to stress, pain stimulating pathways and sex hormone effects on gastrointestinal function [34]. A survey found that rectal discomfort thresholds were significantly lower in female compared to male patients with IBS [35]. The majority of western population studies have reported female-to-male ratios of 2–3∶1 [29], [36]. Studies in Asia including ours showed less dramatic gender difference [37], [38].

We showed IBS of the diarrhea dominant type was more common than the constipation dominant type, similar to a Chinese population study [19]. Dorn and Ersryd also reported IBS-D was the more prevalent subtype, followed by IBS-C [39], [40]. One reason may be that cold water intake may lead to lower visceral perception thresholds in Chinese IBS-D patients [41], [42]. Irregular intake of cold drinks is common in college and university students.

Similar with previous studies, our study shows psychological disturbance is associated with IBS [43]. A recent research provided first direct evidence to indicate that the brain-gut pathway is bidirectional which means both brain-gut and gut-brain dysfunction may be meanwhile occurring in patients of FGIDs [44]. Generally, psychological distress is directly related to the severity and degree of impairment in IBS [45]. The stress and negative life events may affect gastrointestinal function, the pain complaint, quality of life, work absenteeism, health care use and social costs [29], [46], [47].

The prevalence of FC in college students of North China in the present study was 5.45%. Most of the FC patients developed from childhood constipation. Healthy education first trained to children about constipation is inadequate. The children may become scared of the process of defecation and it may lead to the development of chronic constipation into adult [48], [49]. The incidence suggests more attention should be paid in childhood management. Our study also found that most students with FC were affected by the straining involved during defecation and others suffered the sensation of incomplete evacuation. The reason is that the college and university students are young and they usually have irregular eating habits, bedtimes and not enough exercise. Considering the Chinese college and university students as a group within specific age and environmental constraints, this result may be considered rational [50], [51].

The limitation of this study was based solely on the self-reporting questionnaires, without any further examinations necessary to exclude structural intestinal diseases. Moreover, the survey was restricted to a community of college and university students and was not representative of the similar age group. More risk associations like diet, exercise and other psychological factors could be analyzed in future study.

In conclusion, this is the largest school-based population investigation on the epidemiology of FGIDs using Rome III criteria. Symptoms of FGIDs including FD, IBS and FC are quite prevalent in college and university students of North China, with more female students than male students suffering from FGIDs. Psychological disorders such as anxiety and depression provide significant risk factors for IBS patients.
